# ED_50_ and ED_95_ of intrathecal hyperbaric ropivacaine for parturients undergoing cesarean section with prophylactic infusion of phenylephrine: A Prospective dose-finding Study

**DOI:** 10.1097/MD.0000000000013727

**Published:** 2018-12-14

**Authors:** Wenping Xu, Fei Xiao, Yinfa Zhang, Lin Liu, Xiangyang Chang

**Affiliations:** Department of Anesthesia, Jiaxing University Affiliated Women and Children Hospital, Jiaxing, Zhejiang, China.

**Keywords:** cesarean section, combined spinal-epidural anesthesia, dose-response, phenylephrine, ropivacaine

## Abstract

**Background:**

Studies have reported that the ED_50_ of intrathecal ropivacaine was increased when using prophylactic infusion of phenylephrine to prevent spinal-induced hypotension. However, ED_95_ is more meaningful to clinical practice than ED_50_. Therefore, we conducted this study to determine the 95% effective dose (ED_95_) of intrathecal hyperbaric ropivacaine for cesarean section in parturients receiving prophylactic infusion of phenylephrine to prevent spinal-induced hypotension.

**Methods:**

A hundred of healthy parturients undergoing elective cesarean section under combined spinal-epidural anesthesia (CSEA) were enrolled in this randomized, double-blinded, dose-ranging study. Patients were randomly assigned to receive 7, 9, 11, 13 or 15 mg intrathecal hyperbaric ropivacaine respectively. The prophylactic phenylephrine infusion (50 μg/min) was initiated immediately at the same time of spinal injection. Successful spinal anesthesia was defined as a T5 sensory level achieved within 10 min after intrathecal drug administration and no epidural supplement was required during the surgery. The ED_95_ was calculated with Probit analysis.

**Results:**

The ED_95_ of intrathecal ropivacaine with 5 μg sufentanil for successful anesthesia was 15.2 mg (95%CI, 13.5–18.8 mg), when receiving prophylactic infusion of phenylephrine.

**Conclusion:**

Under the conditions of the present study, the ED_95_ of intrathecal hyperbaric ropivacaine for successful spinal anesthesia for cesarean section in healthy parturient receiving prophylactic infusion of phenylephrine was 15.2 mg.

## 1 Introduction

Spinal anesthesia is widely used in cesarean section because of its reliable effect and rapid onset.^[1]^ High incidence of spinal-induced hypotension is the main limitation of this technique.^[2–4]^ Phenylephrine has been recommended to prevent or treat the spinal-induced hypotension for patients undergoing cesarean section, as its more effective and less fetal academia.^[5–7]^ Interestingly, studies focused on phenylephrine reported that preventive intravenous continuous injection of phenylephrine can decrease the rostral spread of bupivacaine or levo-bupivacaine in pregnancy.^[8–10]^ Moreover, one published study has demonstrated that the ED_50_ of intrathecal ropivacaine for cesarean section with prophylactic infusion of phenylephrine was higher than without prophylactic infusion of phenylephrine.^[11]^ In clinical practice, as we known, 95% effective dose (ED_95_, the dose is sufficient for 95% of patient to achieve effective anesthesia) is more meaningful than ED_50_ for patients undergoing cesarean section. Therefore, in this study, we aimed to determine the ED_95_ of intrathecal hyperbaric ropivacaine for patients undergoing cesarean section with intravenous prophylactic infusion of phenylephrine.

## 2 Methods

### 2.1 Design

This study was approved by the Ethics Committee in Jiaxing University Affiliated Women and Children Hospital (the batch number is 20180018) and all parturients signed the written informed consent. We registered this study in the Chinese Clinical Trial Registry (registration number is ChiCTR1800014620). We designed a prospective, randomized, double-blinded study to determine a dose-response study of intrathecal hyperbaric ropivacaine for cesarean section under spinal anesthesia in healthy patients who received prophylactic phenylephrine to prevent spinal-induced hypotension.

### 2.2 Subjects and setting

Inclusion criteria were healthy parturients with an ASA statue of I or II, single pregnant. Exclusion criteria were patients with obesity (body mass index, BMI > 35 kg/m^2^), gestational age < 37 weeks, active labor, early labor, ruptured membranes, history of previous cesarean deliveries, diabetes or gestational diabetes, hypertension or pre-eclampsia, intrauterine growth restriction, placenta previa, significant coexisting maternal disease, any contraindication to spinal or epidural anesthesia such as local infection or bleeding disorders.

### 2.3 Study protocol

All participants received no premedication. After arriving in operating theater, all patients’ peripheric vein was punctured with an 18G puncture needle, and 37 ° C *Lactate Ringer solutions* were injected slowly just to keep the vein open before the induction of spinal anesthesia. Patients’ electrocardiogram (ECG), non-invasive blood pressure (NIBP), heart rate (HR), oxygen saturation (SpO_2_) were checked and recorded. The average of the first 3 readings was considered as the basal NIBP and HR.

With the parturients in left lateral position, the combined spinal-epidural anesthesia (CSEA) was performed using the needle-through-needle technique. After the interspace of L3–4 was estimated, epidural space was ascertained with the method of loss-of-resistance-to-air technique (the air volume < 2 ml) using an 18-G Tuohy needle. Then a 27-G spinal-needle passed through the Tuohy needle to get to the subarachnoid space. When the flow of the cerebrospinal fluid (CSF) observed, the mixed study solution was administered via the spinal needle over 15 s. Before removed the spinal needle, withdrawing the CSF again making sure the drug was injected into the subarachnoid space. If failed to withdraw the CSF, the subject was excluded from the study. Then the anesthesiologist removed the spinal needle, and inserted an epidural catheter into the epidural space by 3–4 cm. No local anesthetic was given through epidural catheter at the moment. With a position of a 15-degree tilt to the left side, the patients received 500 ml *Lactate Ringer solutions* as a co-load in 20 min.

Parturients were randomized allocated into 1 of 5 groups (Group 1, Group 2, Group 3, Group 4, and Group 5) based on a computer-generated random number list (Microsoft, Excel) which was kept in a sealed opaque envelope before the start of the study. Patients in each group received a study solution containing 7 mg, 9 mg, 11 mg, 13 mg, and 15 mg hyperbaric ropivacaine respectively for spinal anesthesia. The mixed study local anesthetic (containing 0.5 ml of 10% dextrose, different dose of ropivacaine and 5 μg of sufentanil diluted a volume of 3 ml with saline) for spinal anesthesia was prepared in a sterile condition in advance by a fixed anesthesiologist, who was not involved in assessing the effect of anesthesia.

The primary endpoint of this study was effective anesthesia or ineffective anesthesia. The secondary outcomes of this study were the characteristics of spinal anesthesia and side-effects. Effective anesthesia was defined, according to previous report,^[12]^ as a bilateral T5 sensory block level to pinprick was achieved within 10 min of intrathecal drug administration and no epidural supplement required during surgery. While ineffective anesthesia was defined as a T5 sensory level was not obtained within 10 min after drug administration or patients complain of pain during surgery. If ineffective anesthesia happened, 2% lidocaine was administrated through epidural catheter in 5 ml increment with 5 min intervals to obtain a T5 sensory level or to rescue intraoperative pain.

At the same time of spinal injection, an infusion of phenylephrine was initiated at a speed of 15 ml/h (50 μg·min^−1^, 10 mg phenylephrine was diluted with saline in a 50 ml syringe). And the infusion rate was adjusted according to the systolic blood pressure (SBP). If the SBP decreased by more than 20% of the baseline, a bolus of 50 μg phenylephrine was administered and the infusion rate was doubled. If the SBP increased more than 10% of baseline, the infusion rate was halved, and if the SBP increased more than 20% of baseline or over 140 mmHg, which was considered as hypertension and the infusion was stopped.

Postoperative pain was treated by patient-controlled analgesia (PCA) pump, which was set with a bolus of 2 μg sufentanil and 10 min of locking time and with 3 μg/h of background dose.

### 2.4 Measurements

Patients’ demographic data including age, body weight, height, gestational age, and duration of surgery were also recorded.

An average of 3 consecutive measurements at the time when parturients arrived in operating room with a supine position was defined as baseline of NIBP or baseline of HR. A consecutive monitoring of NIBP and HR was applied and recorded the values at 2-min intervals from the beginning of spinal anesthesia to the time of baby delivery and at 5-min intervals thereafter. Hypotension was defined as a systolic arterial pressure below 90 mmHg, or a decrease of more than 20% of basal SBP. Bradycardia, defined as HR less than 55 beats per minute, was treated with 0.5 mg of atropine intravenously.

Sensory block level was checked bilaterally along the midclavicular line with pinprick (patient was asked to report pain sensation, if the block was not even bilaterally, the lower side was chosen). Motor block in the lower limbs was graded by a Bromage Score (0 = able to lift extended leg; 1 = able to flex knee but not lift extended leg; 2 = able to move foot only; and 3 = unable to move foot). Both sensory and motor block was assessed at 2-min intervals in the first 10 min, and then at 10 min intervals during surgery. The epidural supplement of 2% of lidocaine was also recorded. The total dose of phenylephrine was also recorded.

Satisfaction of the operation condition (such as the degree of abdominal muscle relaxation) was assessed by the surgeon who performed the cesarean section, ranked as good, moderate, or poor. Subjective pain was assessed with a visual analogue scale (VAS) ranged from 0 to 10 (0 = no pain, 10 = maximum undesirable pain) at the following time points: skin incision, baby delivery, peritoneal closure, skin closure. After the surgery, patients were required to fill out the satisfaction questionnaire (1 = satisfied; 2 = moderate; 3 = poor).

Side effects such as hypotension, hypertension bradycardic, nausea, and vomiting, shivering, pruritus were recorded and studied. The pH value of umbilical arterial blood which was drawn immediately after infant delivery was assessed as the outcome of the infant.

### 2.5 Statistical analysis

The sample size was calculated by Cochran-Armitage Test using PASS software based on the results of our preliminary study. A total number of 100 (5 groups) is sufficient to achieve 95% power to detect a linear trend using a *Z* test with continuity correction and a significance level of .05. Statistical Analysis was performed with SPSS 13.0 for Windows (SPSS Inc., Chicago, IL, USA). Numerical variables were presented as mean and standard deviation (SD) or median (range) where appropriate. Categorical data (incidence data) were presented as numbers or percentages. Means with normally distributed were analyzed by 1-way analysis of variance (ANOVA), medians and means with non-normally distributed were analyzed by Mann-Whitney *U* test, incidence data were analyzed by Fisher exact test. The ED_50_ and ED_95_ of intrathecal bupivacaine were calculated by a logistic regression model described by Khaw et al^[13]^ and Chen et al^[14]^ previously. Logistic regression was used to identify possible significant factors influencing effective or ineffective anesthesia. Statistical significance was defined as *P* < .05 (2-sided)

## 3 Results

**Figure 1:**
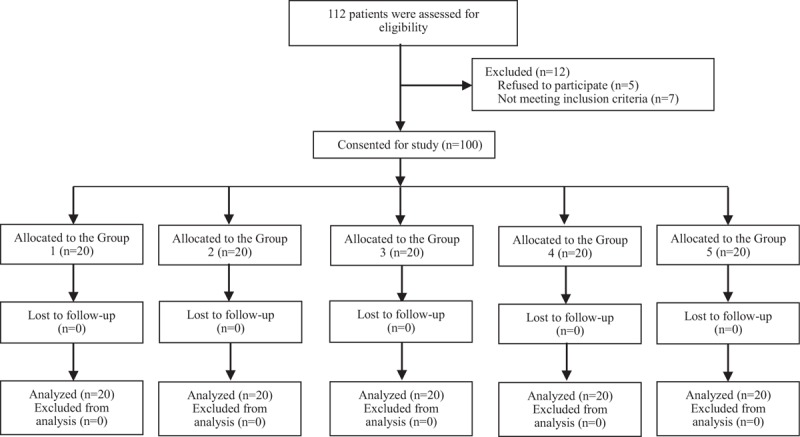
CONSORT Diagram.

The CONSORT diagram is shown in Figure 1. This clinical trial was initiated on 10th June 2017, and was accomplished on 1st Dec 2017. During this period, a 112 parturients were involved and assessed for the suitability in this clinical trial. Finally, 100 of parturients were enrolled and allocated into the 5 groups averagely. And none of the parturients was lost in the final analysis. There is no significant differences in patients’ demographic characteristics (Table 1).

**Table 1:**
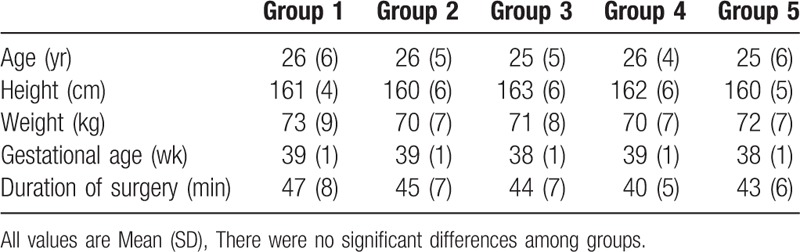
Demographic data and duration of surgery.

**Figure 2:**
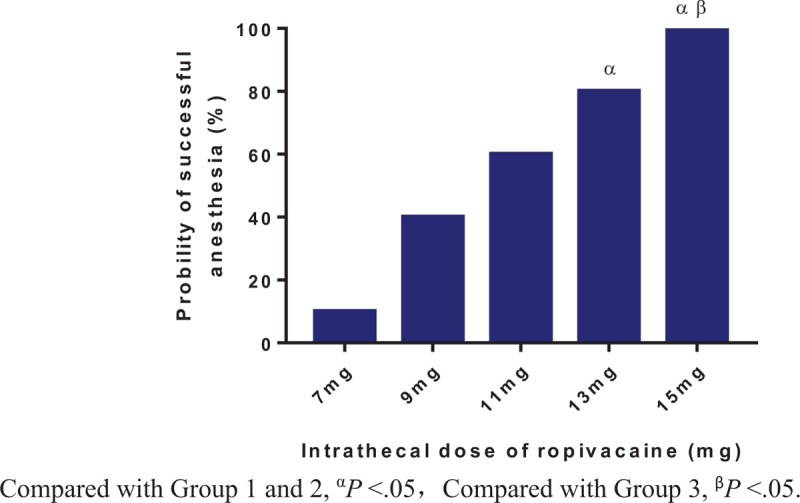
The percentage of effective spinal anesthesia in all patients.

**Figure 3:**
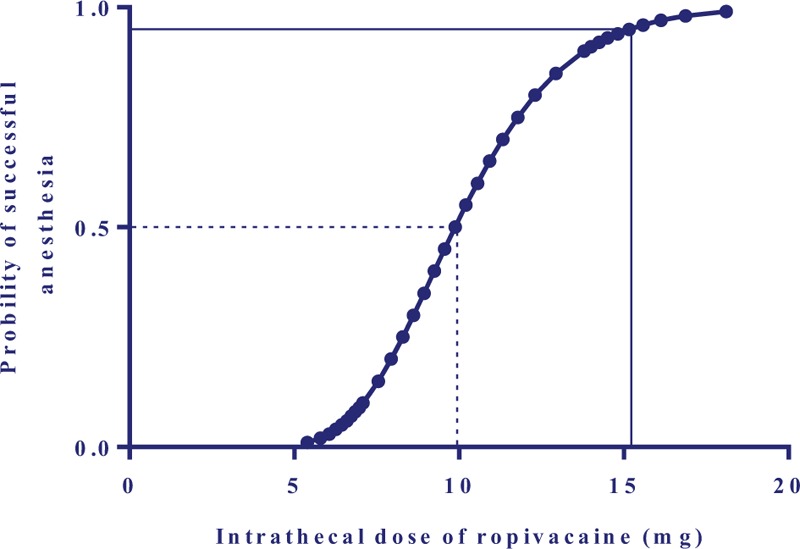
Logistic regression plot represents the probability of effective spinal anesthesia vs intrathecal bupivacaine dose. Probability of 50% (the dotted line) and 95% (the solid line) were used for deriving the ED_50_ and ED_95_ of intrathecal ropivacaine to achieve effective spinal anesthesia for C-section.

The percentages of effective spinal anesthesia are shown in Figure 2. The success anesthesia rate was higher in Group 4 and 5 when compared to Group 1 and Group 2 (*P* < .05). Logistic regression plots were drawn for successful spinal anesthesia, which are presented in Figure 3. The ED_50_ and ED_95_ of intrathecal hyperbaric ropivacaine for effective anesthesia were 9.9 mg (95%CI, 9.0–10.7 mg) and 15.2 mg (95%CI, 13.5–18.8 mg) respectively.

Anesthetic characteristics are shown in Table 2. The sensory block level was significantly higher in high-dose groups (Group 4 and 5) than in low-dose groups (Group 1, 2, and 3) 10 min after spinal injection (*P* < .05). There were 18 parturients in Group 1, 12 in Group 2, 8 in Group 3, 4 in Group 4 and none in Group 5 required additional epidural 2% lidocaine. The requirement of rescued 2% lidocaine was similar among groups (*P* > .05) (Table 2). The total dose of phenylephrine used was similar between groups (*P* > .05) (Table 2). The incidences of hypotension, hypertension, shivering nausea and vomiting were also similar among groups (*P* > .05) (Table 2).

**Table 2:**
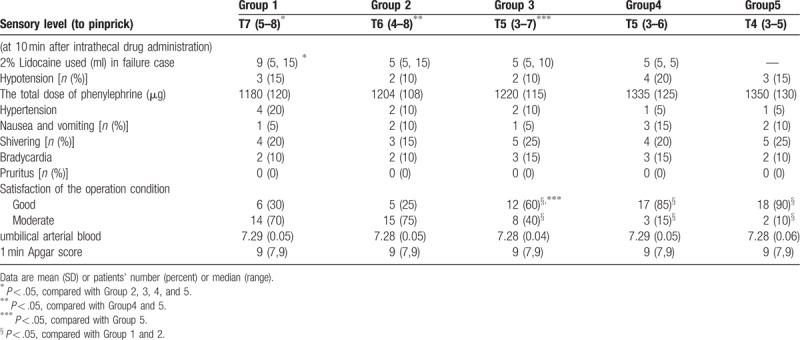
Sensory level and side effects.

**Figure 4:**
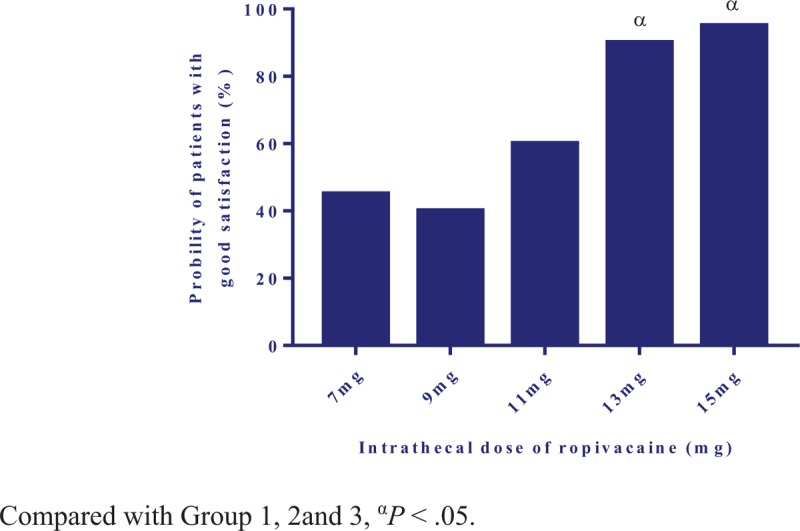
The percentage of patients with good satisfaction.

Satisfaction of the operation condition assessed by surgeon was poorer in Group 1 and Group 2 than Group 4 and Group 5 (*P* < .05) (Table 2). The rate of patient's satisfaction was higher in Group 4 and Group 5 than in Group 1 and Group 2 (*P* < .05) (Fig. 4). There were no significant differences among groups in Apgar scores at 1, 5 min and fetal umbilical artery blood gas analysis (Table 2).

## 4 Discussion

We found that the ED_50_ and ED_95_ of intrathecal ropivacaine for cesarean section were 9.9 mg (95%CI, 9.0–10.7 mg) and 15.2 mg (95%CI, 13.5–18.8 mg) respectively for parturients received prophylactic infusion of phenylephrine for preventing spinal-induced hypotension.

Studies^[8–10]^ have been reported that phenylephrine infusion can decrease the rostral spread of intrathecal local anesthetic in patients undergoing cesarean section. Moreover, Zhang et al compared the ED_50_ of intrathecal ropivacaine when with or without phenylephrine infusion, and found a higher dose requirement when using phenylephrine infusion to prevent spinal-induced hypotension.^[11]^ However, the optimum dose for clinical practice is 95% effective dose, which can meet 95% of patients’ analgesia requirement, rather than ED_50_. Additionally, phenylephrine is regarded as the first-line vasopressor for preventing spinal-induced hypotension in cesarean section. Therefore, it is very necessary to determine the dose requirements of intrathecal local anesthetic for cesarean section when prophylactic phenylephrine infusion is used.

Ngan reported a higher ED_95_ of intrathecal ropivacaine than our results, even without phenylephrine infusion.^[13]^ As we have known, the required dose of spinal anesthetic for cesarean section is influenced by several factors such as the maternal position during performing anesthesia,^[14,15]^ speed of injection of intrathecal solution,^[16]^ gravity of intrathecal solution,^[17]^ race of parturients,^[14]^ co-administration of intrathecal opioids^[18]^ and so on. Between our present study and Ngan et al^[13]^ study, there were several differences in maternal position during performing spinal anesthesia (lateral vs sitting), race, intrathecal opioids (without vs with fentanyl) existed. Therefore, we believed that results would be not comparable between the 2 studies. Fortunately, in Xiao and his colleagues study, whose study protocol was similar to this study, they found ED_50_ and ED_95_ of intrathecal hyperbaric ropivacaine of successful spinal anesthesia (operation) for cesarean section in parturients without receiving prophylactic infusion of phenylephrine were 8.28 mg and 12.24 mg respectively,^[19]^ whereas in our present study the ED_50_ and ED_95_ of intrathecal hyperbaric bupivacaine were 9.9 mg and 15.2 mg respectively, which was higher than Xiao results. By the comparison, it could demonstrate that a higher dose of intrathecal ropivacaine is needed when we choose prophylactic phenylephrine infusion to prevent spinal-induced hypotension.

It is well known that pregnancy leads to the engorgement of epidural venous out of more intra-abdominal pressure, which leads to a decrease of CSF volume in lumbar area.^[9]^ Subsequently, it brings about a decrease in the spinal requirement of local anesthetic or augments in its spinal spread. However, the prophylactic infusion of phenylephrine to prevent post-spinal hypotension may contract the veins in epidural space. Perhaps it can abate the effect of that epidural vein engorgement replace the CSF in lumbar area, and subsequently offset the pregnancy-induced decrease in intrathecal dose requirement. This may be the first mechanism to explain the results of our study. A second possible mechanism would be that phenylephrine, as well as epinephrine, would delay the rise of the spinal block. However, further studies are needed to investigate this suspicion. Previous studies have already reported that intravenous infusion of phenylephrine can affect the spread of spinal local anesthetic (hyperbaric bupivacaine or plain levo-bupivacaine) by 2 segments.^[8,20]^ The clinical significance of this finding remains unknown.

The present study also showed that prophylactic infusion of phenylephrine (50 μg·min^−1^) can improve the stability of the hemodynamics (lower incidence of hypotension), decreased nausea and vomiting. Although low-dose of intrathecal local anesthetic was recommended as a strategy to prevent spinal-induced hypotension,^[21]^ the shortcomings of this strategy is obvious such as lower score of patient's comfort and shorter duration of anesthesia and analgesia when compared to routine dose. Our present study also showed that the probability of successful anesthesia and patients’ satisfaction with anesthesia were higher in the high-dose group than that in the low-dose group, whereas there was no difference in side effects (such as hypotension) and well being of newborns among groups. Therefore, we strongly recommend using a relative higher-dose (ED_95_ or a little higher) of intrathecal local anesthetic for cesarean section by using the strategy of prophylactic infusion of phenylephrine to prevent hypotension.

Limitations also existed in this study. Firstly, obesity patients were excluded from the current study. Different degree of BMI may affect the dose requirement of intrathecal ropivacaine. And further study should focus on this issue. Secondly, we did not observe the duration of block in the study.

In summary, the ED_50_ and ED_95_ of intrathecal hyperbaric ropivacaine for healthy parturients undergoing cesarean section with CSEA were 9.9 mg (95%CI, 9.0 – 10.7 mg) and 15.2 mg (95%CI, 13.5–18.8 mg) respectively, when prophylactic 50 μg·min^−1^ infusions of phenylephrine was applied for preventing spinal-induced hypotension.

## Acknowledgments

The authors would thank all staffs in the department of anesthesia and operating room of Jiaxing University Affiliated Women and Children Hospital for their help in this study.

## Author contributions

**Data curation:** Ping Wen Xu and Lin Liu.

**Formal analysis:** Fei Xiao.

**Investigation:** Ping Wen Xu, Lin Liu, Fa Yin Zhang, and Yang Xiang Chang.

**Methodology:** Ping Wen Xu, Fei Xiao, and Fa Yin Zhang.

**Writing – original draft:** Fei Xiao.
